# Pharmacists’ views on potential non-medical prescribing for pharmacists in Sweden: a nationwide survey study

**DOI:** 10.1007/s11096-025-02006-x

**Published:** 2025-09-27

**Authors:** Nicole Ljungdahl, Sofia Kälvemark-Sporrong, Albin Tranberg, Thomas Kempen

**Affiliations:** 1 Department of Pharmacy, Uppsala University, Uppsala, Sweden; 2 Utrecht Institute for Pharmaceutical Sciences, Utrecht University, Utrecht, the Netherlands

**Keywords:** Non-medical prescribing, Pharmacists, Surveys and questionnaires

## Abstract

**Introduction:**

The role of pharmacists in healthcare has evolved, and in several countries, they have prescribing rights. Currently, pharmacists in Sweden do not have the right to prescribe. Exploring their views on pharmacist prescribing may be a critical step toward advancing the profession and improving patient care.

**Aim:**

To explore pharmacists’ views on potential pharmacist prescribing in Sweden.

**Method:**

An online questionnaire about pharmacist prescribing was distributed via email to Swedish pharmacists who are members of the Swedish Pharmacists Association (n = 5597). The questionnaire covered demographic information and statements about predefined prescribing models, preconditions, benefits, and risks associated with pharmacist prescribing. Each statement had a 4-point agreement Likert scale response option with additional “Don´t know/no opinion” option. Data were collected in May 2024. Closed-ended questions were analyzed descriptively, while open-ended questions were categorized and summarized.

**Results:**

Of the 395 respondents (7% response rate), 75.2% (n = 297) agreed with introducing at least one model of pharmacist prescribing. The two models that received the most support were “Prescribing based on an agreement or collaboration with one or more independent prescribers” (50.1%; n = 198) and “Independent, but limited prescribing rights” (48.1%; n = 190). All predefined preconditions were important for the respondents; their education (97.2%, n = 384) was identified as the most important factor. Overall, respondents agreed more with the benefits than the risks of introducing pharmacist prescribing. Most frequently (somewhat) agreeing that an enhanced professional position of pharmacists in health care was a key benefit (91.1%; n = 369), and the risks that respondents most (somewhat) agreed with included increased workload (80.8%; n = 319) and the conflict of interest involved in pharmacists acting as both prescribers and dispensers (60.8%; n = 240).

**Conclusion:**

The majority of respondents supported introducing at least one pharmacist prescribing model in Sweden. These findings provide valuable insights for policymakers considering an expanded role for pharmacists in Sweden, which may include a variety of extended professional roles in different settings.

**Supplementary Information:**

The online version contains supplementary material available at 10.1007/s11096-025-02006-x.

## Impact statements

- The positive views on pharmacist prescribing could influence policymakers to introduce expanded rights for pharmacists in the future.
- These findings may act as a starting point for further research on the views of other interested parties such as patients and medical doctors on potential pharmacist prescribing in Sweden.
- Policymakers should carefully plan for an introduction of pharmacist prescribing rights including setting an education standard and clear guidelines on the scope of prescribing rights

## Introduction

Today, the healthcare sector faces growing pressure from a shortage of professionals, such as physicians and nurses, and the rising demand for medical services and medications driven by an aging population [1, 2]. At the same time, there is a profession whose expertise lies in medications –yet their competence remains underutilized: pharmacists. In response to these challenges, pharmacists have expanded their roles in healthcare, from product-oriented to more person-centered care services [3, 4]. Pharmacy services can decrease patients’ adverse drug events, hospitalizations, overall mortality, and quality of life [5–8]. Pharmacists in countries like Canada, Denmark, New Zealand, Poland, the United Kingdom (UK), and the United States of America have prescribing rights to improve care access [1, 9–14]. Evidence suggests that pharmacist prescribing is as effective as prescribing by physicians [15], reduces physicians’ perceived workload, and increases patients’ access to medications [16–18].

The pharmacist prescribing types vary between and within countries [19]. Collaborative prescribing, which can be defined as prescribing conducted within the context of a collaborative team environment or collaborative agreement between a pharmacist and a physician [20], is classified as a dependent prescriber. In New Zealand, pharmacists who are part of a multidisciplinary healthcare team can initiate or modify treatment for specific patients and conditions [9]—Independent prescribing, where pharmacists have full autonomy to prescribe medication exists in various forms; in the UK, independent prescribing rights for pharmacists include the ability to prescribe for any condition, whether diagnosed or undiagnosed. Pharmacists in this context are responsible for making clinical decisions, including prescribing medications, a role similar to that of physicians [10, 21]. In other countries, independent prescribing may be more restricted, such as being limited to prescribing for minor ailments, for example, in some parts of Canada and Denmark [13, 14, 22].

Despite evidence and examples from other countries, Sweden has not adopted prescribing rights for pharmacists. However, pharmacists have increasingly been integrated into the healthcare system, particularly in secondary and primary care settings, and some pharmacy services are available to a limited degree in community pharmacies. The primary role of clinical pharmacists is to perform medication reviews in patients with polypharmacy [23–25] and contribute with pharmaceutical expertise to the healthcare team, hence collaborating with prescribers [25, 26]. Whether Swedish pharmacists want to obtain prescribing rights, what prescribing model would be suitable, under what conditions, and with what benefits for patients and healthcare, is essential to understand before pursuing such rights [16, 27, 28]. Given the growing interest in pharmacist prescribing and the evolving role of pharmacists within healthcare systems, understanding Swedish pharmacists’ views on potential prescribing rights is crucial in shaping future policy discussions.

### Aim

To explore Swedish pharmacists’ views on potential pharmacist prescribing in Sweden.

## Method

### Study design and population

A national cross-sectional online survey was conducted in Sweden in May 2024. The population consisted of “prescriptionists” (*receptarier* in Swedish) who had obtained a Bachelor of Science (BSc.) in Pharmacy, and “pharmacists” (*apotekare*) with a Master of Science (MSc.) in Pharmacy [29]. In 2022, there were 11,493 employed registered pharmacists in Sweden, 5687 with a BSc. degree and 5806 with an MSc. degree [30]. Many pharmacists are members of the Swedish Pharmacists Association trade union, with just over 7100 members in 2024. Of these, 2919 worked in community pharmacy, 1072 in industry, 1225 in the public sector (including hospitals, primary care centers, and authorities), 381 in other sectors, and 1503 were student members (Personal communication, the Swedish Pharmacists Association). All prescriptionists and pharmacists, regardless of work setting, and excluding students, were considered potential participants in this study.

### Questionnaire development

The questionnaire was developed in several steps. The initial version was created in Swedish based on previous research [16, 27, 31] and was pilot-tested among Swedish clinical pharmacists in 2022 [32]. It was translated into English and further developed through discussions involving 17 pharmacists and researchers from various European countries and Taiwan during a workshop at the Pharmaceutical Care Network Europe (PCNE) conference in Denmark, in February 8–11, 2023. The revised version was back-translated into Swedish using deepl.com, and adapted to the Swedish context [33]. The questionnaire was further piloted with three pharmacists working in various settings in Sweden (public healthcare, community pharmacy, and authority) with a think-aloud method [34]. This was to assess its understandability in different work sectors. Minor adjustments were made, resulting in 15 questions, including the first question to request the participant´s consent (Online Resource 1). A final pilot test of the questionnaire was conducted with four pharmacists in academia to verify technical functionality, spelling, and the time required (see Online Resource 1 for more information about the participants). After the pilot tests, the questionnaire was estimated to take 15–20 min to complete.

**Fig. 1:**
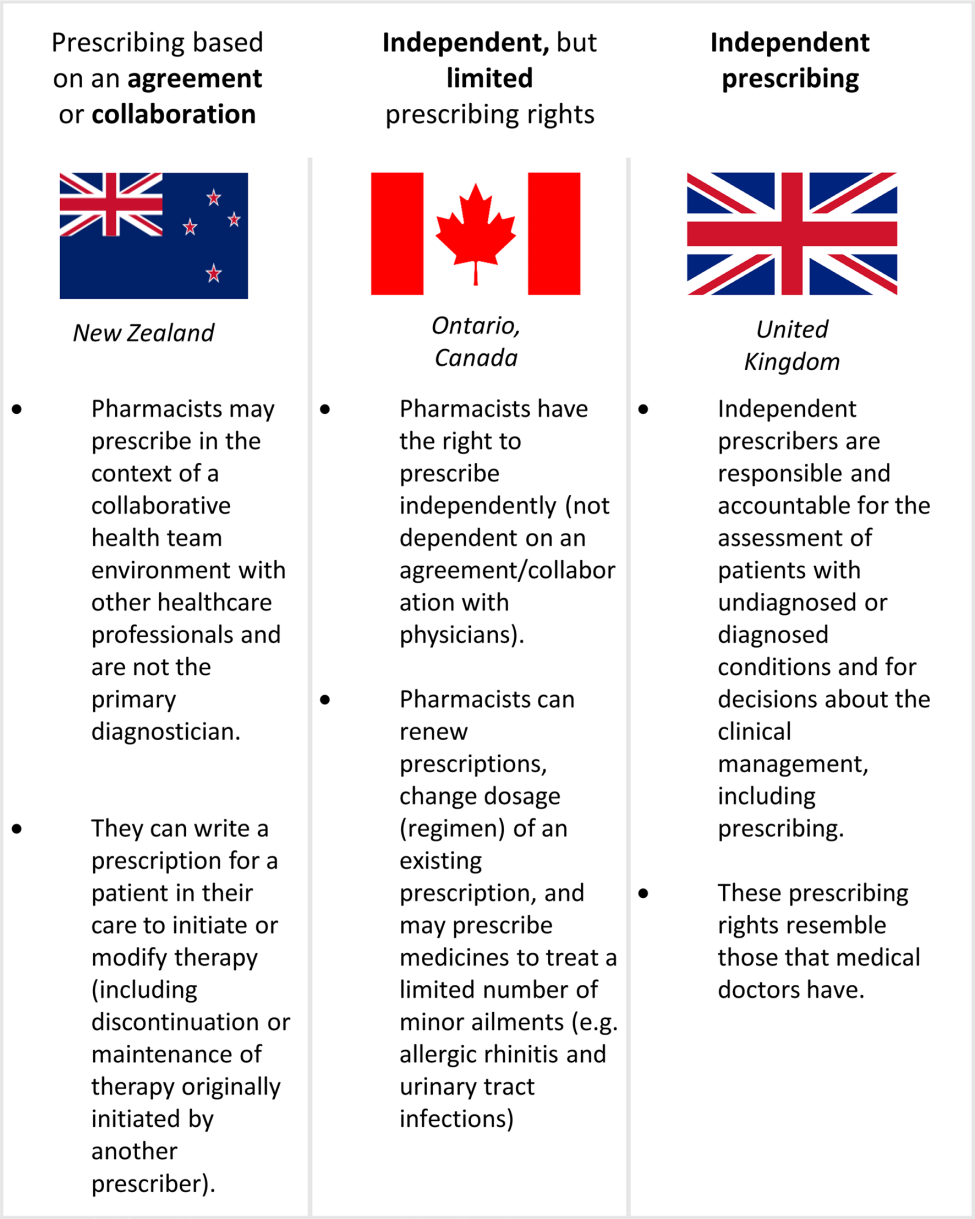
Models of prescribing rights for pharmacists from different countries (New Zealand, Ontario (Canada) and the United Kingdom) that were presented as examples to the survey participants

The questionnaire included demographic questions and views on pharmacist prescribing, including four models based on examples from other countries (Fig. 1). Additionally, it included questions about settings, preconditions for implementing prescribing rights, and the benefits and risks of pharmacist prescribing. Apart from the demographic questions, all questions were formulated as statements with a Likert scale (1 = Disagree; 2 = Somewhat disagree; 3 = Somewhat agree; 4 = Agree; and 0 = Don´t know/no opinion) as response options [35]. Respondents who selected “Disagree” regarding introducing any prescribing model automatically skipped other questions related to the prescribing models.

### Recruitment and data collection

The link to the questionnaire was distributed via email to all members of the Swedish Pharmacists Association, excluding students (n = 5597). Based on a power calculation, a study population of 370 respondents was required to achieve 95% confidence level. Study data were collected and managed using Research Electronic Data Capture (*REDCap*), hosted at Uppsala University [36, 37]. REDCap is a secure, web-based software platform that supports data capture for research studies. The questionnaire was open for three weeks in May 2024, with a reminder sent after two weeks. The questionnaire link was open-access, allowing anyone to share it without informing the researchers.

### Data analysis

Data were exported to the IBM Statistical Package for Social Sciences (SPSS) for analysis. Respondents were excluded if they lacked a pharmacist degree, did not consent, or did not complete the entire questionnaire. To avoid duplicates from the open-access link, incomplete responses were excluded. Data were analyzed using descriptive statistics to get an overview of the results. Open-text comments were thematically categorized based on the items in the questionnaire addressing prescribing rights.

Primary and secondary healthcare settings were merged into one group: “clinical healthcare”, due to their comparable roles and contextual similarities, as well as the limited number of respondents in each setting. This category also includes respondents working in healthcare and other settings. Community pharmacy was treated as a separate setting due to its large proportion among the respondents. This category also includes respondents who work in community pharmacy alongside any of the “other” settings. All other respondents, working in authorities or industry, were merged into an “other” group.

Subgroup analyses were conducted for education, work experience, and work settings. Non-parametric tests were applied because normal data distribution could not be assumed. Mann–Whitney U-tests were used to analyze differences based on education level and work experience. To minimize the number of subgroups, respondents with an MSc. in pharmacy or an MSc. in clinical pharmacy (a one-year MSc. degree) were combined into the “MSc. degree” group, and respondents’ work experience was categorized into < 1 year to 4 years and ≥ 5 years. The Kruskal–Wallis test was used to analyze the difference between work settings. P-values lower than 0.05 were considered significant. Bonferroni corrections were applied to account for multiple testing.

The study is reported following the guidelines outlined in the Consensus-Based Checklist of Reporting of Survey Studies (CROSS) [38].

### Ethics approval

The Swedish Ethics Review Authority approved the study in December 2023 (Reg.no.: 2023–04602-01).

## Results

Out of the 5597 pharmacists invited, 527 initiated the questionnaire, and 395 met the inclusion criteria (7% response rate) (Online Resource 2). Among the respondents, 81.5% (n = 322) were women and 42.8% (n = 169) held a BSc. degree (Table 1). Most worked in community pharmacy, 50.4% (n = 201) and 80.5% (n = 318) had five or more years of work experience.

**Table 1:** Demographic information for the respondents who completed the entire questionnaire (n = 395)

Characteristic		N (%)
Gender	Women	322 (81.5)
	Men	69 (17.5)
	Non-binary	0 (0.0)
	Do not want to answer	4 (1.0)
Highest finished degree	Bachelor of Science in Pharmacy	169 (42.8)
	Master of Science in Pharmacy	226 (55.2)
Work setting	Community pharmacy	201 (50.4)
	Clinical healthcare ^a^	78 (17.5)
	Other ^b^	116 (29.4)
Length of work experience	< 1 year to 4 years	77 (19.5)
	≥ 5 years	318 (80.5)
Patient contact in work practice	Daily	211 (53.4)
	Weekly	29 (7.3)
	Monthly	11 (2.8)
	More rarely	54 (13.7)
	Never	90 (22.8)
Age, median years (min–max)		46 (25–83)

^a^Clinical healthcare includes primary care and/or hospital care

^b^Other includes industry, authorities, and other settings, e.g., consultant, pensioner

### Prescribing models

#### Agreement with models of prescribing

**Fig. 2:**
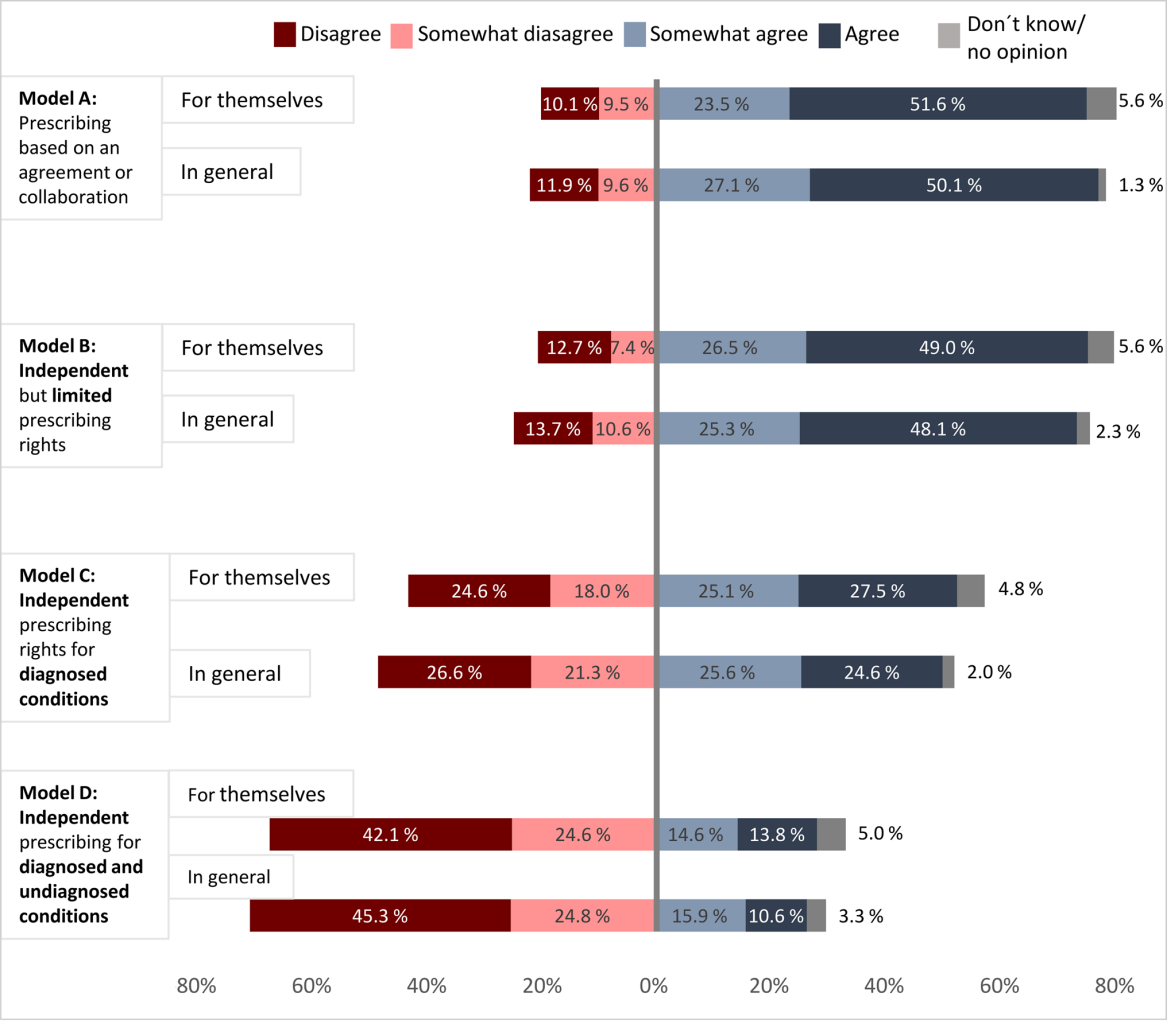
Respondents’ agreement with future prescribing rights (n = 395). “For themselves” = To what extent respondents are willing to have the following prescribing rights in the future for themselves. “In general” = To what extent respondents agreed that the following models of prescribing rights for pharmacists should be introduced in Sweden, in general

Overall, 75.2% (n = 297) of the respondents agreed with introducing at least one of the proposed prescribing models, and 91.6% (362) agreed or somewhat agreed with at least one of the proposed models. The most favored models, based on the proportion of “agree” responses were “Prescribing based on an agreement or collaboration with one or more independent prescribers” (model A; 50.1%; n = 198) and “Independent, but limited prescribing rights” (model B; 48.1%; n = 190; Fig. 2). Additionally, 24% (n = 97) agreed with “Independent prescribing rights in patients with diagnosed conditions” (model C), while 10.6% (n = 42) supported “Independent prescribing rights in both patients with diagnosed and undiagnosed conditions” (model D). In general, similar results were observed when respondents answered which prescribing rights they were willing to have themselves (Fig. 2). Seventeen (4.5%) respondents, of whom 13 worked in community pharmacy, disagreed with all the proposed prescribing models. Based on their comments, the main reasons for this were a lack of time for pharmacists and a perceived lack of competence.

**Fig. 3:**
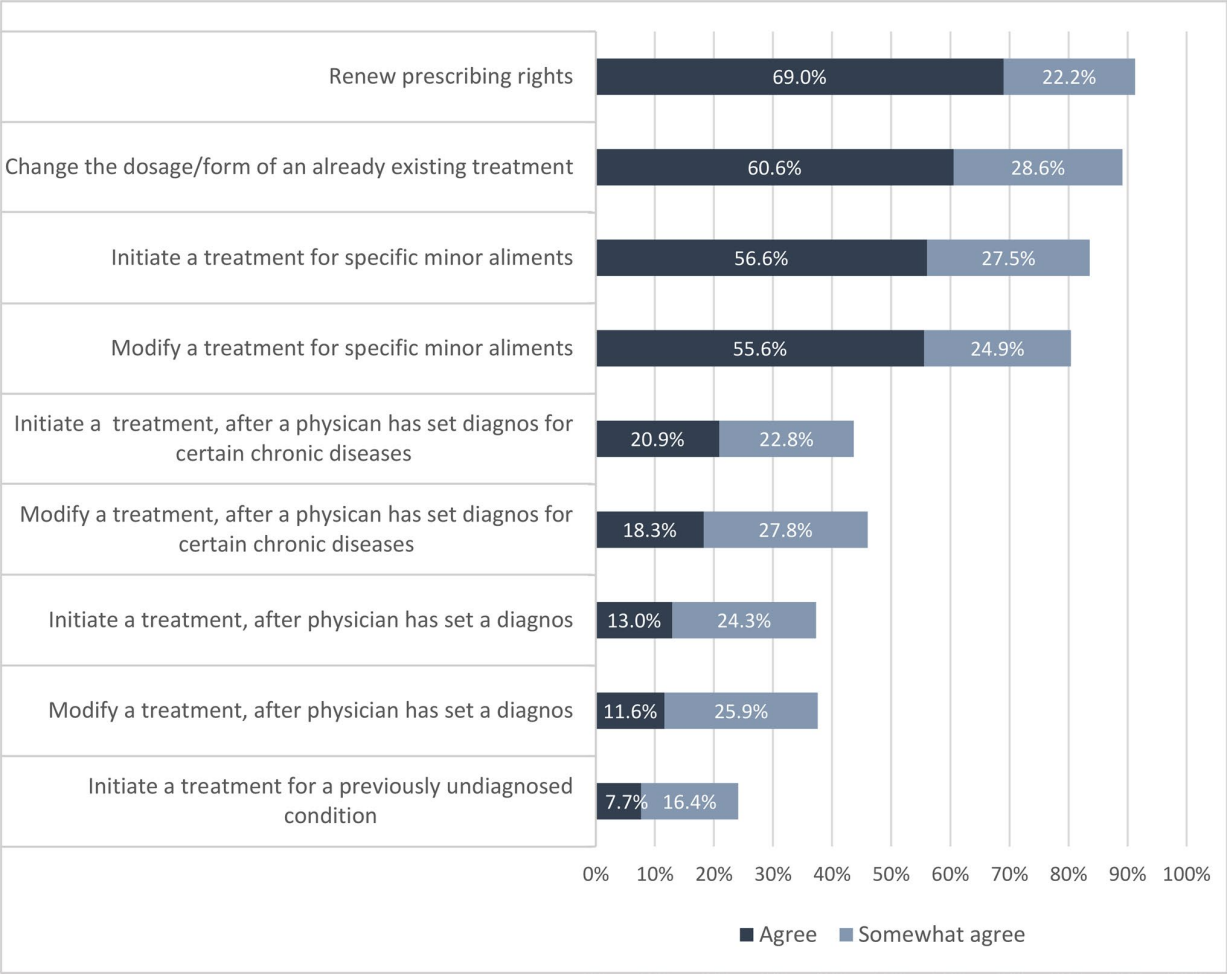
Agreement with prelisted tasks respondents are willing to have if pharmacist prescribing is introduced in Sweden (n = 395)

Furthermore, respondents were particularly positive about the tasks that would mostly be performed in models A and B. These tasks include the ability to renew prescriptions, modify the dosage or form of existing treatments, and initiate or modify therapy for specific minor ailments, as reflected in both prelisted options (80.4%-91.3% agreed; Fig. 3) and the open-text comments. Moreover, respondents were less positive about more complex tasks that would be needed for models C and D, such as modifying or initiating treatment for certain chronic diseases and modifying or initiating treatment, regardless of disease, diagnosed or undiagnosed (24.1%-43.7% agreed).

#### Subgroup analyses

Regardless of work setting, respondents seemed more supportive towards introducing prescribing based on a collaborative agreement (model A) and independent but limited prescribing rights (model B) compared to the other models. Respondents working in community pharmacies expressed less support for introducing independent prescribing for patients with diagnosed conditions than those in “other” settings (p-value: < 0.001). No differences were observed in support for the other models among pharmacists from different work settings (see Online Resource 2 for detailed information).

Respondents with an MSc. degree showed greater support for the independent prescribing models (model B-D) (p-value: < 0.001)*.* Overall, respondents with ≥ 5 years of work experience were generally less supportive of implementing any of the prescribing models in Sweden, with significant differences in support for models B and D (p-values: 0.036 and 0.022, respectively).

### Settings to introduce pharmacist prescribing

Support for (agree or somewhat agree with) pharmacist prescribing appeared substantial across all four prelisted settings: primary care (84.4%; n = 319), tertiary care (81.2%; n = 307), secondary care (75.4%; n = 285), and community pharmacy (75.2%; n = 284) (Online Resource 2). However, (80%; n = 152) of respondents working in community pharmacy also supported introducing it in their own setting. The main reasons for not supporting the introduction of prescribing rights in community pharmacy – according to free text comments – were the belief that pharmacists do not have a comprehensive view of a patient´s health and treatment, and a lack of time.

### Preconditions for introducing pharmacist prescribing

**Fig. 4:**
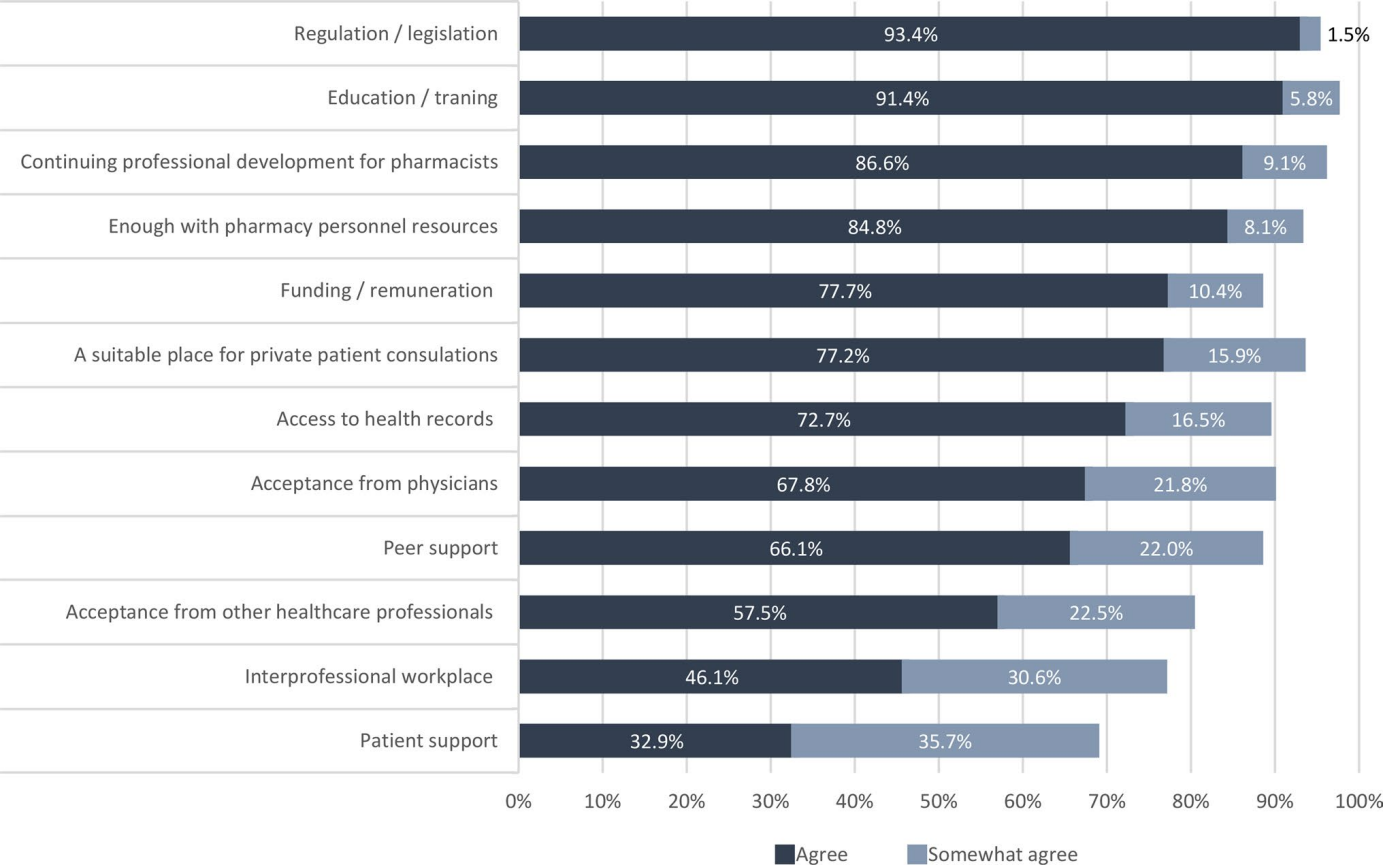
Agreement with prelisted preconditions to introduce pharmacist prescribing, presented as the percentage of respondents who agreed or somewhat agreed (n = 395)

A set of preconditions was included in the questionnaire (Fig. 4). For all prelisted conditions, the majority of respondents (somewhat) agreed that they should be fulfilled. The highest level of agreement was for education/training (97.2%; n = 384), continuing professional development for pharmacists (95.7%; n = 378), and regulation/legislation (94.9%; n = 375). Acceptance from physicians seemed more important (89.6%; n = 354) compared to other healthcare professions (80.0%; n = 316) or patients (68.6; n = 271). In addition to the listed preconditions, respondents also mentioned in free text the importance of having work experience, better communication systems between healthcare professionals, and holding an MSc. as opposed to a BSc. degree as important factors.

### Pharmacists’ views on benefits and risks of pharmacist prescribing

**Fig. 5:**
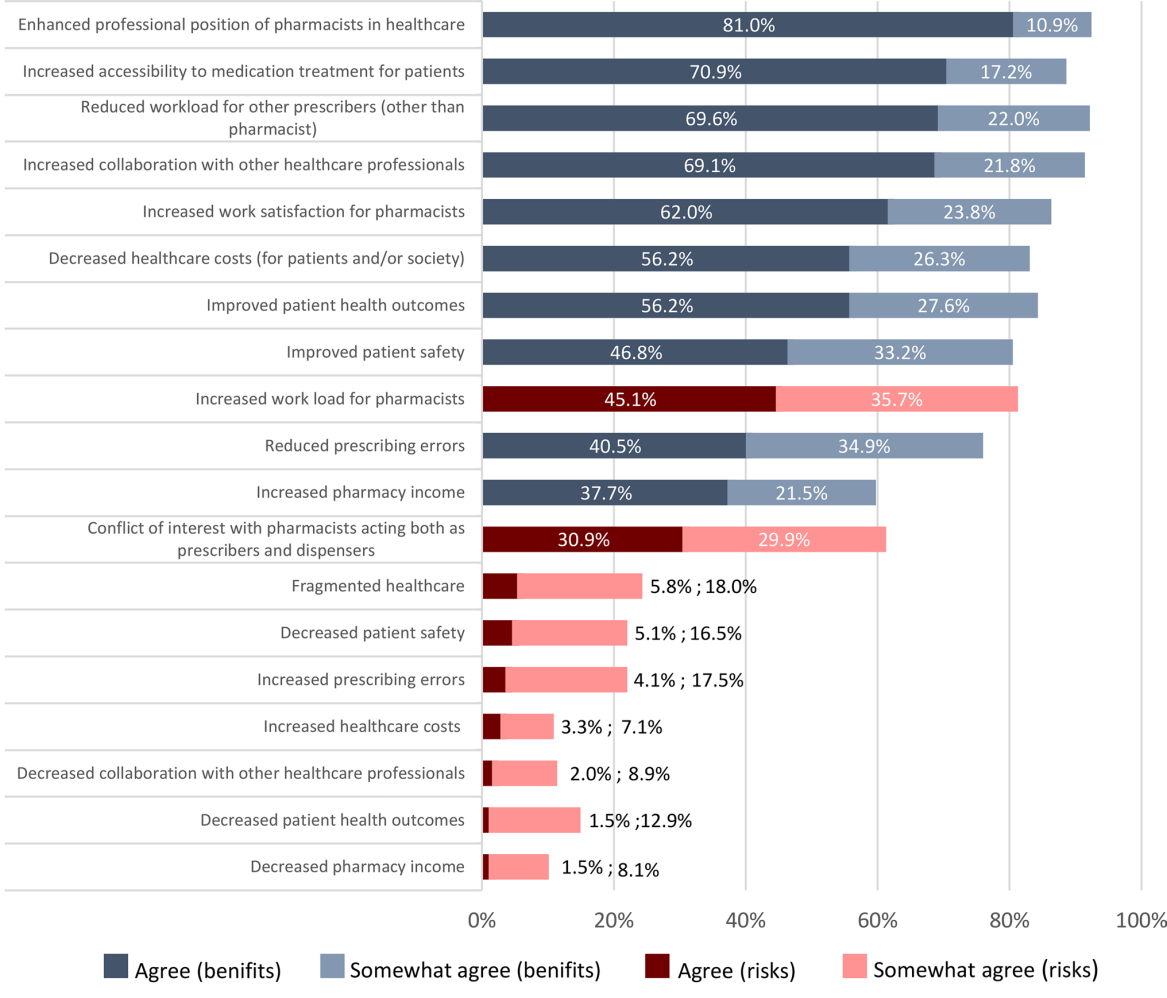
Respondents’ views on the benefits and risks of introducing pharmacist prescribing, presented as the percentage of respondents who agreed or somewhat agreed with a given benefit or risk (n = 395). Dark and light blue represent the benefits, while red and pink represent the risks

Overall, respondents were more likely to (somewhat) agree with the listed benefits than with the risks. The three most positively viewed benefits were the enhanced professional position of pharmacists in healthcare (91.9%; n = 363), reduced workload for other prescribers (91.6%; n = 362), and increased collaboration with other healthcare professionals (90.9%; n = 359). The risks that respondents most (somewhat) agreed with included increased workload and the conflict of interest involved in pharmacists acting as both prescribers and dispensers (Fig. 5)*.* Additional risks mentioned in free text included increased threats from patients seeking more medication, and the perception of pharmacist prescribing as a profit-driven venture for pharmacies, which could lead to medication overprescribing.

## Discussion

This study explored pharmacists’ views on potential pharmacist prescribing models in Sweden. Most respondents either agreed or somewhat agreed with the idea of introducing prescribing rights for pharmacists, whether in a collaborative or independent but limited capacity (e.g., for specific patient groups or minor ailments). This was also reflected in a high level of agreement with prescribing for less complex tasks, such as renewing prescriptions, modifying the dosage or form of existing treatments, and initiating or modifying therapy for specific minor ailments. There was a high level of agreement with most prelisted preconditions and there seemed greater agreement with predefined benefits than risks associated with introducing pharmacist prescribing. However, two risks were particularly noteworthy: an increased workload for pharmacists, and a potential conflict of interest when pharmacists act both as prescribers and dispensers.

### Strengths and weaknesses

The questionnaire was developed in several stages with input from international academics and pharmacists. The Swedish version was adapted and pilot-tested several times to ensure clarity and relevance. However, some limitations need to be addressed. Due to the open-access link to the questionnaire, it is not certain that all respondents are members of the Swedish Pharmacists Association. However, what is essential for the study is to gather the views of Swedish pharmacists on prescribing rights. The study had a low response rate, extended data collection and additional reminders might possibly have increased the response rate. Nevertheless, the demographic characteristics of the respondents aligned with Sweden´s national pharmacist distribution in terms of gender and education level [30]. The small sample size and unequal subgroup sizes make it difficult to draw reliable conclusions about between-group differences [39]. Pharmacists who supported expanding the role of pharmacists in healthcare may have been more inclined to participate, possibly leading to an overrepresentation of positive views. Nonetheless, some respondents expressed opposition to pharmacist prescribing. The use of predefined models, along with a list of preconditions, risks, and benefits derived from the literature, may have influenced or limited the respondents’ answers to the questionnaire. Furthermore, it remains unclear whether respondents answered questions about preconditions, risks, and benefits with a specific model in mind.

### Interpretation

The positive views towards pharmacist prescribing among Swedish pharmacists align with previous studies on pharmacists' prescribing in other countries [16, 40]. The model of independent prescribing within a limited scope (model B), which was a highly supported prescribing model, is similar to the current prescribing rights held by certain nurse practitioners in Sweden. Since 1994, nurses who have completed specific educational programs have had independent prescribing rights for limited conditions and medications [41, 42].

Respondents generally viewed models A and B positively, endorsing some form of dependency on an existing treatment by a physician and less complex prescribing rights for tasks such as renewing prescriptions, adjusting dosages, or modifying drug formulations. These rights are seen as incremental changes that would not necessitate significant systemic adjustments, especially in community pharmacy settings. Community pharmacists in Sweden only have the right to correct technical errors in prescriptions related to the patient's handling and use of medicines. Expanded rights could enhance time efficiency by minimizing the need to contact prescribers regarding dosage issues, formulation adjustments, or replacements of unavailable medications.

Respondents with a BSc. degree showed a more conservative perspective on the future of pharmacist prescribing in Sweden compared to those with advanced education. This suggests that a three-year BSc. degree might not provide sufficient competence for prescribing medications. Nonetheless, previous studies indicate that pharmacists agree with the necessity of additional specialized education before prescribing rights can be granted [16]. Research from New Zealand, Australia, Canada, and the UK highlights the importance of sufficient and relevant education in fostering the confidence and competence necessary for pharmacists to prescribe medications [43].

In contrast to a similar survey study in the Netherlands [40], respondents in this study identified community pharmacies as the least likely setting for pharmacist prescribing. This may result from the limited regular communication between Swedish community pharmacists and other healthcare providers, which makes the transition to prescribing seem more challenging. In contrast, pharmacists in clinical healthcare settings often collaborate closely with physicians and have access to patients’ medical records [24].

Furthermore, most respondents believed that acceptance from physicians is essential. This perspective is highlighted by the fact that the development of independent prescribing rights for nurses in Sweden has been slow compared to other countries [44]. Several factors likely contribute to this, including divided opinions among physicians regarding the role of nurses [45, 46] and differences in perspective between nurses and physicians [47]. If pharmacist prescribing is to be possible, it is important to include physicians’ views to foster a better understanding and collaboration.

Most respondents believed that pharmacists’ workload would increase, despite already being substantial. There is currently a significant pharmacist shortage in Sweden, especially in community pharmacies [48], which may have influenced their responses. Another risk identified was the potential conflict of interest when pharmacists serve as both prescribers and dispensers, a concern that previous studies have addressed [49]. However, in the UK, community pharmacists with prescribing rights seem to prescribe medication less frequently compared to pharmacists in collaborative healthcare settings [50].

## Conclusion

Most respondents supported introducing at least one pharmacist prescribing model in Sweden, indicating a promising foundation for potential future implementation. Simpler tasks, such as renewing prescriptions, adjusting dosages, or modifying drug formulations, could lead to a more time-effective approach for both pharmacists and physicians. However, further research is needed to include the perspectives of other interested parties, e.g., patients and physicians. Several preconditions must be met to ensure successful implementation, including access to medical records, sufficient financial resources, and adequate time. A possible initial step for prescribing rights could be to introduce it for clinical pharmacists in hospital and primary care settings, where structures for collaboration and interprofessional support are already in place.

## Supplementary Information

Below is the link to the electronic supplementary material.

Supplementary file1 (PDF 441 KB)

Supplementary file2 (PDF 378 KB)

## Data Availability

The datasets generated during and/or analysed during the current study are available from the corresponding author on reasonable request.
