# Supplementary material for: Pharmacists’ views on potential non-medical prescribing for pharmacists in Sweden: a nationwide survey study
Source: Int J Clin Pharm. 2025 Sep 27;48(2):501–12. doi: 10.1007/s11096-025-02006-x (PMC12992373; doi:10.1007/s11096-025-02006-x)
Supplement: Supplementary file 1 — Supplementary file1 (PDF 441 KB) [file 11096_2025_2006_MOESM1_ESM.pdf]

Nicole Ljungdahl1*, Sofia Kälvemark-Sporrong1, Albin Tranberg1, Thomas Kempen1,2. Affiliations: 1. Department of Pharmacy, Uppsala, Sweden, 2. Utrecht Institute for Pharmaceutical Sciences, Utrecht, the Netherlands. *Email: nicole.ljungdahl@uu.se

Supplementary 1 - Additional information about the questionnaire

Information about the participants for pilot testing the questionnaire

In pilot test two, we attempted to recruit pharmacists from various work settings to explore the questionnaire's understandability from different perspectives. The second aim was to see the time required. For pilot test three, the objective was to test the technological, design, spelling, and the time required.

Table 1: Demographic information about the participants for the pilot tests two and three.

**Pilot-test 2: think-aloud**				
**ID**	**Gender**	**Education**	**Work setting**	**Work experience (years)**
**1**	Woman	Master of Science in Pharmacy	Public healthcare sector	2
**2**	Woman	Master of Science in Pharmacy	Authority	1.5
**3**	Woman	Master of Science in Pharmacy	Community pharmacy	3
**Pilot-test 3: technical and spelling test**				
**1**	Woman	Master of Science in Pharmacy	Academic - teacher	5
**2**	Woman	Master of Science in Pharmacy	Academic - teacher	Over 5
**3**	Woman	Bachelor of Science in Pharmacy	Academic - teacher	Over 5
**4**	Woman	Bachelor of Science in Pharmacy	Academic - Project assistant	3

An information letter for the survey was distributed by the Swedish Pharmacists Association (trade union).

Research in the field of pharmacy is important to us and can, for example, demonstrate the benefits that the profession brings to society. One topical issue is pharmacist prescribing; we must support research in this area. We would therefore like to ask you to participate in a survey.

The Swedish Pharmacists Association is only helping to distribute this anonymous survey to you and is not responsible for the survey or the answers. If you choose to answer the survey, your answers will be sent directly to the research group from the Department of Pharmacy at Uppsala University. The survey is independent of the Swedish Pharmacists' membership and completely voluntary. The purpose of the survey is described below.

IP addresses may be collected in survey tools to facilitate participation. By answering the survey, you consent to the sharing of this personal data.

About the survey

We, a research group from the Department of Pharmacy, Uppsala University, would like to ask you to participate in a research project. Below is information about the project and what it means to participate.

Why is this project relevant and why do you want me to participate?

Prescribing by pharmacists, pharmacist prescribing, is practiced in some countries, such as the UK, Northern Ireland, Denmark, and New Zealand. However, the regulation and scope of pharmacist prescribing differ between countries.

It is important to know pharmacists' views on the issue of pharmacist prescribing in Sweden to support future actions by policymakers, educators, and other stakeholders. The aim of the project is to gain insight into how pharmacists working in Sweden view pharmacist prescribing, and we would like you, as a pharmacist, to participate in the study

How does the study work?

Participation involves answering an online survey that takes about 15-20 minutes. The survey is mainly about your views on pharmacists' prescribing rights. The survey is sent out in several European countries, including Sweden, the Netherlands and Belgium.

The survey is distributed via the Swedish Pharmacists Association on our behalf, but the survey responses will only be processed by the research team. Your participation is anonymous and we cannot access any personal data. Your participation is voluntary and you agree to participate by answering and submitting the survey. We want to receive your response by May 27.

How will I be informed about the results of the study?

The study results will be published in a scientific article and a popular form. You can find out about the results by contacting the people responsible for the study.

The survey is available here: XXXX

If you have any questions about the survey, please contact one of the researchers. Thank you in advance for your participation!

NAMES AND E-MAIL ADDRESSES OF THE RESEARCHERS

Nicole Ljungdahl1*, Sofia Kälvemark-Sporrong1, Albin Tranberg1, Thomas Kempen1,2. Affiliations: 1. Department of Pharmacy, Uppsala, Sweden, 2. Utrecht Institute for Pharmaceutical Sciences, Utrecht, the Netherlands. *Email: nicole.ljungdahl@uu.se

The full questionnaire

	**Translate English version (original in Swedish)**
**intro**	Below is some brief information about the questionnaire content that you need to read before you answer the questions.<br><br>The survey contains questions about your views on pharmacist prescribing. It will take approximately 15-20 minutes to complete. All responses are anonymous and no answers can be related to any specific individual. Your participation is voluntary and you agree to participate by completing and submitting the survey.<br><br>*Terms used in the survey*<br><br>*Pharmacist prescribing:* prescribing medicines within the pharmacist's clinical competence based on a professional pharmaceutical assessment of the patient, including taking responsibility for the prescribed treatment and documenting the process.<br><br>*Patients:* this includes both patients and pharmacy customers.
1.	Are you:<br>- Male<br>- Female<br>- Other<br>- Don't want to answer
2	Year of birth**:**<br>______________________________
3	What pharmacist education(s) have you completed:<br><br>- Bachelor of Pharmacy<br>- Master of Pharmacy<br>- Master in clinical pharmacy<br>- Other pharmacy degree:_____<br>- No pharmacy degree
4	For how long have you been practicing as a pharmacist?<br>- < 1 year<br>- 1-2 years<br>- 3-4 years<br>- 5 or more years
5	Within which setting are you currently practicing? You can give multiple answers. –<br>• Community pharmacy (including internet pharmacies)<br>• Primary care (e.g. general practice)<br>• Secondary care (hospital or specialty care)<br>• Industry<br>• Government authority

<table>
<tr><td></td><td>Other:______</td></tr>
<tr><td>6</td><td>How often do you have direct patient contact in your current work practice?
- Daily
- Weekly
- Monthly
- Rarely
- Never</td></tr>
<tr><td colspan="2">
The next questions concern your perspective on different potential models of pharmacist prescribing in Sweden. Below you will find examples of models that have been implemented in other countries.
<table>
<tr><td>Prescribing based on an agreement or collaboration</td><td>Independent, but limited prescribing rights</td><td>Independent prescribing</td></tr>
<tr><td>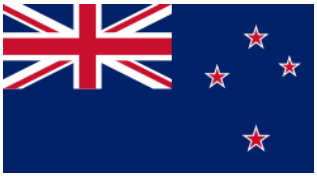
New-Zealand</td><td>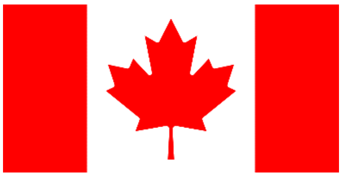
Ontario, Canada</td><td>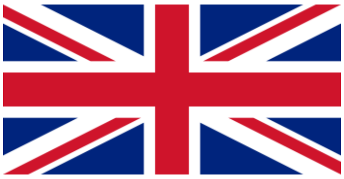
United Kingdom of Great Britain</td></tr>
<tr><td>• Pharmacists may prescribe in the context of a collaborative health team environment with other healthcare professionals and are not the primary diagnostician.</td><td>• Pharmacists have the right to prescribe independently (not dependent on an agreement/collaboration with physicians).</td><td>• Independent prescribers are responsible and accountable for the assessment of patients with undiagnosed or diagnosed conditions and for decisions about the clinical management, including prescribing.</td></tr>
<tr><td>• They can write a prescription for a patient in their care to initiate or modify therapy (including discontinuation or maintenance of therapy originally initiated by another prescriber).</td><td>• Pharmacists can renew prescriptions, change dosage (regimen) of an existing prescription, and may prescribe medicines to treat a limited number of minor ailments (e.g. allergic rhinitis and urinary tract infections)</td><td>• These prescribing rights resemble those that medical doctors have.</td></tr>
</table>
In all prescribing models, pharmacists can only prescribe within the limits of their professional expertise and competence.
</td></tr>
<tr><td>Answer options</td><td>- Disagree
- Somewhat disagree</td></tr>
</table>

**for questions 7-13**	- Somewhat agree<br>- Agree<br>- Don't know / No opinion
7	To what extent do you agree that the following models of prescribing rights for pharmacists should be introduced in Sweden?<br><br>a. Prescribing based on an **agreement** or **collaboration** with one or more independent prescribers (e.g. physicians). This agreement could be about prescribing for a single patient or certain patients/situations in general.<br><br>b. **Independent** (of a physician), but **limited** prescribing rights (e.g. specific patient groups/health conditions/drug list/formulary)<br><br>c. **Independent** prescribing rights in patients with **diagnosed conditions** (being responsible and accountable for the assessment of the patient and for decisions about the clinical management, including prescribing)<br><br>d. **Independent** prescribing rights in both patients with **diagnosed and undiagnosed conditions** (being responsible and accountable for the assessment of the patient and for decisions about the clinical management, including prescribing)<br><br>Other model(s) of prescribing rights or comments:__________<br><br>*If the participant answers "disagree" on all models at question 7, a new question is asked: "What is the most important reason why you do not want to introduce prescription rights for pharmacists in Sweden?"*
8	To what extent do you agree that pharmacist prescribing would be suitable in the following settings, if introduced in Sweden?<br><br>a. Community pharmacy<br>b. Primary care (e.g. general practice)<br>c. Secondary care (hospital or specialty care)<br>d. Tertiary care (e.g. nursing home)<br><br>Comment:______________
9	To what extent would **you** be willing to have the following prescribing rights **in the future**?<br><br>a. Prescribing based on an **agreement** or **collaboration** with one or more independent prescribers (e.g., physicians). This agreement could be about prescribing for a single patient or certain patients/situations in general.<br><br>b. Independent (of a physician), but **limited** prescribing rights (e.g., specific patient groups/health conditions/drug list/formulary)

Nicole Ljungdahl1*, Sofia Kälvemark-Sporrong1, Albin Tranberg1, Thomas Kempen1,2. Affiliations: 1. Department of Pharmacy, Uppsala, Sweden, 2. Utrecht Institute for Pharmaceutical Sciences, Utrecht, the Netherlands. *Email: nicole.ljungdahl@uu.se

	c. **Independent** prescribing rights in patients with **diagnosed conditions** (being responsible and accountable for the assessment of the patient and for decisions about the clinical management, including prescribing)<br><br>d. **Independent** prescribing rights in both patients with **diagnosed and undiagnosed conditions** (being responsible and accountable for the assessment of the patient and for decisions about the clinical management, including prescribing)<br><br>Other model(s) of prescribing rights or comments___________
10	If **you** would get prescribing rights, to what extent would you agree to do the following?<br><br>a. Renew prescriptions (i.e. renew or repeat an already existing treatment/prescription)<br><br>b. Change the dosage (form) of an already existing treatment/prescription (e.g., higher/lower dose, other time of the day, or other type of inhaler)<br><br>c. Modify a treatment for specific minor ailments (e.g., hay fever)<br><br>d. Initiate a treatment for specific minor ailments (e.g., hay fever)<br><br>e. Modify a treatment, after a physician has set a diagnosis for certain chronic diseases (e.g., hypertension, diabetes or COPD)<br><br>f. Initiate a treatment, after a physician has set a diagnosis for certain chronic diseases (e.g. hypertension, diabetes or COPD)<br><br>g. Modify a treatment, after a physician has set a diagnosis<br><br>h. Initiate a treatment, after a physician has set a diagnosis.<br><br>i. Initiate a treatment for a previously undiagnosed condition (which you, as a pharmacist, diagnose).<br><br>Other options or comments______<br><br>:
11	To what extent do **you** agree that the following conditions are important for the introduction of pharmacist prescribing in Sweden?<br><br>a. Education/Training for pharmacists (related to prescribing)<br><br>b. Continuing Professional Development for pharmacists (a formal system or framework that involves the tracking and documenting of skills, knowledge and experience gained, beyond any initial education and training)

Nicole Ljungdahl1*, Sofia Kälvemark-Sporrong1, Albin Tranberg1, Thomas Kempen1,2. Affiliations: 1. Department of Pharmacy, Uppsala, Sweden, 2. Utrecht Institute for Pharmaceutical Sciences, Utrecht, the Netherlands. *Email: nicole.ljungdahl@uu.se

	c. Patient support (e.g., that patient organisations are supportive)<br><br>d. Peer support (i.e. that the pharmacist professional body is supportive)<br><br>e. Acceptance from physicians<br><br>f. Acceptance from other healthcare professionals (e.g., nurses)<br><br>g. Access to health records<br><br>h. A suitable place for private patient consultations<br><br>i. Interprofessional workplace (e.g., co-location with physicians)<br><br>j. Enough pharmacy personnel resources<br><br>k. Funding/Remuneration<br><br>l. Regulation/Legislation that enables pharmacist prescribing<br><br>Other condition(s) or comments:__________<br><br>____
12	To what extent do you agree that the following benefits would be the result of pharmacist prescribing?<br><br>a. Increased accessibility to medicines treatment for patients<br><br>b. Improved patient health outcomes<br><br>c. Improved patient safety<br><br>d. Reduced prescribing errors<br><br>e. Decreased healthcare costs (for patients and/or society)<br><br>f. Reduced workload for other prescribers (other than pharmacists)<br><br>g. Increased collaboration with other healthcare professionals<br><br>h. Enhanced professional position of pharmacists in health care<br><br>i. Increased pharmacy income<br><br>j. Increased work satisfaction for pharmacists<br><br>Other benefits or comments:<br><br>____

Nicole Ljungdahl1*, Sofia Kälvemark-Sporrong1, Albin Tranberg1, Thomas Kempen1,2. Affiliations: 1. Department of Pharmacy, Uppsala, Sweden, 2. Utrecht Institute for Pharmaceutical Sciences, Utrecht, the Netherlands. *Email: nicole.ljungdahl@uu.se

13	To what extent do you agree that the following risks would be the result of pharmacist prescribing?<br><br>a. Conflict of interest with pharmacists acting both as prescribers and dispensers<br><br>b. Decreased patient health outcomes<br><br>c. Decreased patient safety<br><br>d. Increased prescribing errors<br><br>e. Increased healthcare costs (for patients and/or society)<br><br>f. Increased work load for pharmacists<br><br>g. Decreased collaboration with other healthcare professionals<br><br>h. Fragmented healthcare<br><br>i. Decreased pharmacy income<br><br>Other risks or comments: __________<br><br>____
14	Please write any other comments you have on pharmacist prescribing and relating issues:_____________<br><br>____
