# Supplementary material for: Pharmacists’ views on potential non-medical prescribing for pharmacists in Sweden: a nationwide survey study
Source: Int J Clin Pharm. 2025 Sep 27;48(2):501–12. doi: 10.1007/s11096-025-02006-x (PMC12992373; doi:10.1007/s11096-025-02006-x)
Supplement: Supplementary file 2 — Supplementary file2 (PDF 378 KB) [file 11096_2025_2006_MOESM2_ESM.pdf]

Pharmacists' views on prescribing rights for pharmacists in Sweden: A nationwide survey study. *International Journal of Clinical Pharmacy*.
Nicole Ljungdahl1*, Sofia Kälvemark-Sporrong1, Albin Tranberg1, Thomas Kempen1,2. Affiliations: 1. Department of Pharmacy, Uppsala, Sweden, 2. Utrecht Institute for Pharmaceutical Sciences, Utrecht, the Netherlands. *Email: nicole.ljungdahl@uu.se

Supplementary 2 – Additional survey results

Flowchart of participant inclusion/exclusion

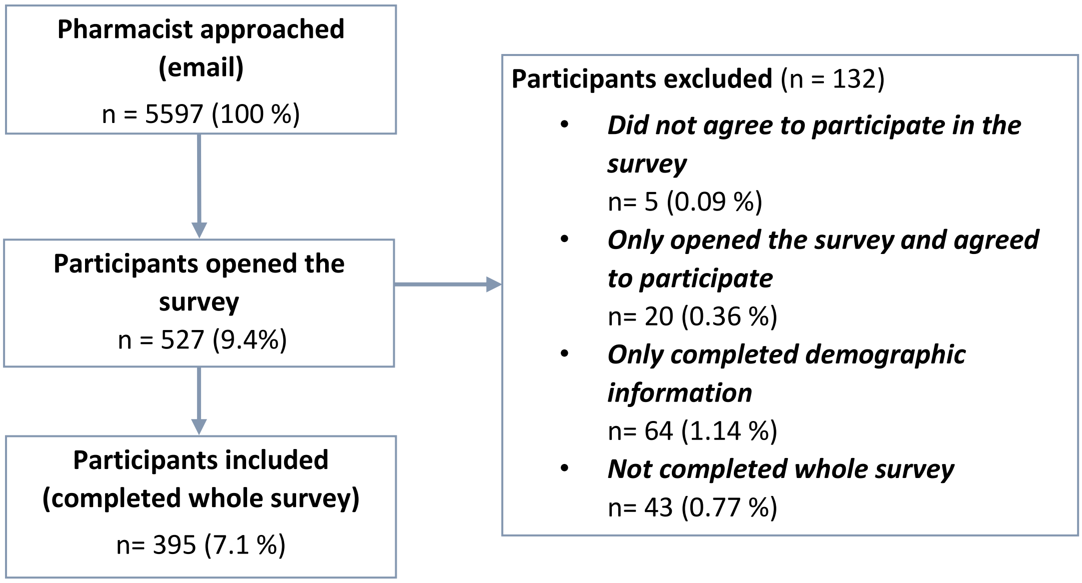

Figure 1: Flowchart of inclusion and exclusion for the study population. "Not completed the whole survey" means that the respondents have started to fill in the questions about prescribing rights, but have not finished all the questions.

Nicole Ljungdahl1*, Sofia Kälvemark-Sporrong1, Albin Tranberg1, Thomas Kempen1,2. Affiliations: 1. Department of Pharmacy, Uppsala, Sweden, 2. Utrecht Institute for Pharmaceutical Sciences, Utrecht, the Netherlands. *Email: nicole.ljungdahl@uu.se

Prescribing models

Respondents' answers to questions about introducing prescribing rights in Sweden and what extent they were willing to have the prescribing models in the future (Table S1). Prelisted work setting as the respondents agree to (Table S2).

Table. S1 Respondents' agreement with future prescribing rights. Presented in percent and number

		Responses % (n)					
Statements		Disagree	Somewhat disagree	Somewhat agree	Agree	Don´t know/ no opinion	Total n
Prescribing based on an agreement or collaboration with one or more independent prescribers	General	11.9 (47)	9.6 (38)	27.1 (107)	50.1 (198)	1.3 (5)	395
	By themselves	*10.6 (38)*	*10.1 (36)*	*24.9 (89)*	*54.5 (195)*	*5.6 (20)*	*378*
Independent (of a physician), but limited prescribing rights	General	13.7 (54)	10.6 (42)	25.3 (100)	48.1 (190)	2.3 (9)	395
	By themselves	*12.7 (48)*	*7.4 (28)*	*26.5 (100)*	*48.9 (184)*	*4.5 (17)*	*378*
Independent prescribing rights in patients with diagnosed conditions	General	26.6 (105)	21.3 (84)	25.6 (101)	24.6 (97)	2.0 (8)	395
	By themselves	24.6 (93)	18.0 (68)	25.1 (95)	27.5 (104)	*4.8 18*	*378*
Independent prescribing rights in both patients with diagnosed and undiagnosed conditions	General	45.3 (179)	24.8 (98)	15.9 (63)	10.6 (42)	3.3 (13)	395
	By themselves	*42.1 (159)*	*24.6 (93)*	*14.6 (55)*	*13.8 (51)*	*5.0 (19)*	*378*

Table. 2 Respondents' agreement with future prescribing rights in prelisted work settings. Presented in percent and numbers

	Disagree % (n)	Somewhat disagree % (n)	Somewhat agree % (n)	Agree % (n)	Don´t know % (n)
Primary care (e.g. general practice)	3.7 (14)	7.4 (28)	23.3 (88)	61.1 (231)	4.5 (17)
Tertiary care (e.g. nursing home)	6.1 (23)	7.7 (29)	27.0 (102)	54.2 (205)	5.0 (19)
Secondary care (hospital or specialty care)	10.6 (40)	8.7 (33)	22.5 (85)	52.9 (200)	5.3 (20)
Community pharmacy	11.1 (42)	12.4 (47)	27.8 (105)	47.4 (179)	1.3 (5)

Nicole Ljungdahl1*, Sofia Kälvemark-Sporrong1, Albin Tranberg1, Thomas Kempen1,2. Affiliations: 1. Department of Pharmacy, Uppsala, Sweden, 2. Utrecht Institute for Pharmaceutical Sciences, Utrecht, the Netherlands. *Email: nicole.ljungdahl@uu.se

Subgroup analysis – Work section

Subgroup analysis is based on the work section and the respondents' agreement to introduce pharmacist prescribing models, and based on the work section and the respondents' agreement to have prescribing rights by themselves (Table S3).

Table. S3 Difference in agreements with prescribing rights based on work sector, presented in numbers and percentages. "By themselves" = To what extent are respondents willing to have the following prescribing rights in the future. "General" = To what extent respondents agreed that the following models of prescribing rights for pharmacists should be introduced in Sweden. Non-parametric, Kruskal-Wallis test with a 95 % confidence interval. Pairwise comparisons of the subgroups and adjusted value using Bonferroni correction for multiple tests. Missing data: 17 respondents who disagreed with all pharmacist prescribing models skipped these questions related to prescribing models. Respondents who answered don´t know or had no opinion were excluded

			**Responses % (n)**						
**Statements**		**Work section**	***Disagree***	***Somewhat disagree***	***Somewhat agree***	***Agree***	***Total***	***Mean***	***P-value***
**Prescribing based on an agreement or collaboration**	In general	***Community pharmacy***	14.1 (28)	12.6 (25)	27.3 (54)	46.0 (91)	100.0 (198)	3.00	0.065
		Health care	6.4 (5)	10.3 (8)	25.6 (20)	57.7 (45)	100.0 (78)	3.35	
		Other	12.3 (14)	4.4 (5)	28.9 (33)	54.4 (62)	100.0 (114)	3.25	
	For themselves	***Community pharmacy***	9.8 (18)	12.5 (23)	26.6 (49)	51.1 (94)	100.0 (184)	3.19	0.238
		Health care	2.7 (2)	8.1 (6)	29.7 (22)	59.5 (44)	100.0 (74)	3.46	
		Other	18 (18)	7.0 (7)	18 (18)	57 (57)	100.0 (100)	3.14	
**Independent (of a physician), but limited prescribing rights**	In general	***Community pharmacy***	18.3 (36)	9.6 (19)	24.4 (48)	47.7 (94)	100.0 (197)	3.02	0.234
		Health care	9.1 (7)	16.9 (13)	28.6 (22)	45.5 (35)	100.0 (77)	3.10	
		Other	9.8 (11)	8.9 (10)	26.8 (30)	54.5 (61)	100.0 (112)	3.26	
	For themselves	***Community pharmacy***	11.4 (21)	9.7 (18)	25.4 (47)	53.5 (99)	100.0 (185)	3.21	0.432
		Health care	13.7 (10)	11 (8)	31.5 (23)	43.8 (32)	100.0 (73)	3.05	
		Other	16.7 (17)	2 (2.0)	29.4 (30)	52 (53)	100.0 (102)	3.17	
**Independent prescribing rights in patients with diagnosed conditions**	In general	***Community pharmacy***	32.3 (63)	24.6 (48)	23.6 (46)	19.5 (38)	100.0 (195)	2.30	**<.001 ***
		Health care	29.9 (23)	18.2 (14)	27.3 (21)	24.7 (19)	100.0 (77)	2.47	
		Other	16.5 (19)	19.1 (22)	29.6 (34)	34.8 (40)	100.0 (115)	2.83	
	For themselves	***Community pharmacy***	28.1 (52)	22.2 (41)	23.8 (44)	25.9 (48)	100.0 (185)	2.48	0.123
		Health care	25.0 (18)	18.1 (13)	29.2 (21)	27.8 (20)	100.0 (72)	2.60	
		Other	22.3 (23)	13.6 (14)	29.1 (30)	35.0 (36)	100.0 (103)	2.77	
**Independent prescribing rights in both patients with diagnosed and undiagnosed conditions**	In general	***Community pharmacy***	49.2 (95)	23.3 (45)	16.6 (32)	10.9 (21)	100.0 (193)	1.89	0.227
		Health care	51.9 (40)	26.0 (20)	10.4 (8)	11.7 (9)	100.0 (77)	1.82	
		Other	39.3 (44)	29.5 (33)	20.5 (23)	10.7 (12)	100.0 (112)	2.03	
	For	***Community pharmacy***	42.9 (79)	29.3 (54)	14.1 (26)	13.6 (25)	100.0 (184)	1.98	0.775
		Health care	47.2	27.8	9.7	15.3	100.0	1.93	

Nicole Ljungdahl1*, Sofia Kälvemark-Sporrong1, Albin Tranberg1, Thomas Kempen1,2. Affiliations: 1. Department of Pharmacy, Uppsala, Sweden, 2. Utrecht Institute for Pharmaceutical Sciences, Utrecht, the Netherlands. *Email: nicole.ljungdahl@uu.se

	themselves		(34)	(20)	(7)	(11)	(72)		
		Other	45.1 (46)	18.6 (19)	21.6 (22)	14.7 (15)	100.0 (102)	2.06	

* Significant difference between two groups.

Pharmacists' views on prescribing rights for pharmacists in Sweden: A nationwide survey study. *International Journal of Clinical Pharmacy*.
Nicole Ljungdahl1*, Sofia Kälvemark-Sporrong1, Albin Tranberg1, Thomas Kempen1,2. Affiliations: 1. Department of Pharmacy, Uppsala, Sweden, 2. Utrecht Institute for Pharmaceutical Sciences, Utrecht, the Netherlands. *Email: nicole.ljungdahl@uu.se

Subgroup analysis – Education

Subgroup analysis is based on education level and the respondents' agreement to introduce pharmacist prescribing models, and based on education level and the respondents' agreement to have prescribing rights by themselves (Table S4).

Table. S4 Difference in agreements with prescribing rights in general and by themselves based on education, presented in both numbers and percentages. Non-parametric, Mann-Whitney U-test with 95 % confidence interval. Missing data: 17 respondents who disagreed with all pharmacist prescribing models skipped these questions related to prescribing models. Respondents who answered "don´t know or had no opinion were excluded.

			Responses % (n)						
**Models**	Sub group	**Level**	***Disagree***	***Somewhat disagree***	***Somewhat agree***	***Agree***	***Total***	***Mean***	***P-value***
**Prescribing based on an agreement or collaboration**	In general	*BS. degree*	12.2 (20)	7.9 (13)	30.5 (50)	49.4 (81)	100.0 (164)	3.17	0.877
		MSc.degreer[a]	11.9 (27)	11.1 (25)	25.2 (57)	51.8 (117)	100.0 (226)	3.17	
	For themselves	*BS. degree*	8.7 (13)	13.3 (20)	24.7 (37)	53.3 (80)	100.0 (150)	3.23	0.768
		MSc.degreer[a]	12.0 (25)	7.7 (16)	25 (52)	55.3 (115)	100.0 (208)	3.24	
**Independent (of a physician), but limited prescribing rights**	In general	*BS. degree*	22.2 (36)	13.6 (22)	24.1 (39)	40.1 (65)	100.0 (162)	2.82	**<.001 ***
		MSc.degreer[a]	8.0 (18)	8.9 (20)	27.2 (61)	55.8 (125)	100.0 (224)	3.31	
	For themselves	*BS. degree*	16.1 (24)	12.8 (19)	28.2 (42)	43.0 (64)	100.0 (149)	2.98	**0.003 ***
		MSc.degreer[a]	11.3 (24)	4.2 (9)	27.4 (58)	57.1 (121)	100.0 (212)	3.30	
**Independent prescribing rights in patients with diagnosed conditions**	In general	*BS. degree*	39.9 (65)	28.8 (47)	22.1 (36)	9.2 (15)	100.0 (163)	2.01	**<.001 ***
		MSc.degreer[a]	17.9 (40)	16.5 (37)	29.0 (65)	36.6 (82)	100.0 (224)	2.84	
	For themselves	*BS. degree*	37.1 (56)	23.2 (35)	26.5 (40)	13.2 (20)	100.0 (151)	2.16	**<.001 ***
		MSc.degreer[a]	17.7 (37)	15.8 (33)	26.3 (55)	40.2 (84)	100.0 (209)	2.89	
**Independent prescribing rights in both patients with diagnosed and undiagnosed conditions**	In general	*BS. degree*	60.4 (99)	23.8 (39)	10.4 (17)	5.5 (9)	100.0 (164)	1.61	**<.001 ***
		MSc.degreer[a]	36.7 (80)	27.1 (59)	21.1 (46)	15.1 (33)	100.0 (218)	2.15	
	For themselves	*BS. degree*	56.0 (84)	28.7 (43)	12.0 (18)	3.3 (5)	100.0 (150)	1.63	**<.001 ***
		MSc.degreer[a]	35.9 (75)	23.9 (50)	17.7 (37)	22.5 (47)	100.0 (209)	2.27	

* Significant difference between two groups.

[a] MSc. in pharmacy or an MSc. in clinical pharmacy (a one-year MSc. degree)

Nicole Ljungdahl1*, Sofia Kälvemark-Sporrong1, Albin Tranberg1, Thomas Kempen1,2. Affiliations: 1. Department of Pharmacy, Uppsala, Sweden, 2. Utrecht Institute for Pharmaceutical Sciences, Utrecht, the Netherlands. *Email: nicole.ljungdahl@uu.se

Subgroup analysis – Work experience

Subgroup analysis is based on working experience and the respondents' agreement to introduce pharmacist prescribing models, and based on experience and the respondents' agreement to have prescribing rights by themselves (Table S5).

Table. S5 Difference in agreements with prescribing rights in general and by themselves based on work experience, presented in both numbers and percentages. Non-parametric, Mann-Whitney U-test with 95 % confidence interval. Missing data: 17 respondents who disagreed with all pharmacist prescribing models skipped these questions related to prescribing models. Respondents who answered "don´t know or had no opinion were excluded

			**Responses % (n)**						
**Models**		Experience	***Disagree***	***Somewhat disagree***	***Somewhat agree***	***Agree***	***Total***	***Mean***	***P-value***
**Prescribing based on an agreement or collaboration**	In general	*<1 to 4 years*	9.1 (7)	3.9 (3)	31.2 (24)	55.8 (43)	100.0 (77)	3.34	0.146
		≥5 years	12.8 (40)	11.2 (35)	26.5 (83)	49.5 (155)	100.0 (313)	3.13	
	For themselves	*<1 to 4 years*	12.2 (9)	2.7 (2)	21.6 (16)	63.5 (74)	100.0 (74)	3.45	0.101
		≥5 years	10.2 (29)	12.0 (34)	25.7 (73)	52.1 (148)	100.0 (284)	3.20	
**Independent (of a physician), but limited prescribing rights**	In general	*<1 to 4 years*	5.3 (4)	11.8 (9)	25.0 (19)	57.9 (44)	100.0 (76)	3.36	**0.036***
		≥5 years	16.1 (50)	10.6 (33)	26.1 (81)	47.1 (146)	100.0 (310)	3.04	
	For themselves	*<1 to 4 years*	8.1 (6)	5.4 (4)	20.3 (15)	66.2 (49)	100.0 (74)	3.45	**0.005***
		≥5 years	14.6 (42)	8.4 (24)	29.6 (85)	47.4 (136)	100.0 (287)	3.10	
**Independent prescribing rights in patients with diagnosed conditions**	In general	*<1 to 4 years*	20.8 (16)	24.7 (19)	28.6 (22)	26 (20)	100.0 (77)	2.60	0.363
		≥5 years	28.7 (89)	21.0 (65)	25.5 (79)	24.8 (77)	100.0 (310)	2.46	
	For themselves	*<1 to 4 years*	23 (17)	21.6 (16)	27.0 (20)	28.4 (21)	100.0 (74)	2.61	0.855
		≥5 years	26.6 (76)	18.2 (52)	26.2 (83)	29.0 (83)	100.0 (286)	2.58	
**Independent prescribing rights in both patients with diagnosed and undiagnosed conditions**	In general	*<1 to 4 years*	36.5 (27)	25.7 (19)	23.0 (17)	14.9 (11)	100.0 (74)	2.16	**0.022***
		≥5 years	49.4 (152)	25.6 (79)	14.9 (46)	10.1 (31)	100.0 (308)	1.86	
	For themselves	*<1 to 4 years*	37.0 (27)	27.4 (20)	15.1 (11)	20.5 (15)	100.0 (73)	2.19	0.101
		≥5 years	46.2 (132)	25.5 (73)	15.4 (44)	12.9 (37)	100.0 (286)	1.95	

* Significant difference between two groups.
